# Prolapsed Bowel Treated by Subcutaneous Strychnia Injections—Dolbean

**Published:** 1861-08

**Authors:** 


DOLBEAN reports two cases of prolapsed bowel, (one in a girl three years old, the other in a boy five years old,) both of which were cured by the subcutaneous injection of sulphate of strychnia, 30 centigrammes to 30 grammes of water. The canula was introduced one centimetre to the right of the anus to the depth of 0.5 centimetre, and 10-11 drops of the solution injected.

